# Adducin in tumorigenesis and metastasis

**DOI:** 10.18632/oncotarget.17173

**Published:** 2017-04-18

**Authors:** Cong Luo, Jiayu Shen

**Affiliations:** ^1^ Zhejiang Cancer Hospital, Department of Abdominal oncology, Hangzhou, Zhejiang, China; ^2^ Zhejiang Chinese Medical University, The Second Clinical Medical College, Hangzhou, Zhejiang, China

**Keywords:** adducin, phosphorylation, tumor

## Abstract

Adducin is a membrane-skeletal protein localized at spectrin-actin junctions, involving in the formation of the network of cytoskeleton, cellular signal transduction, ionic transportation, cell motility and cell proliferation. While previous researches focused mainly on the relationship between adducin and hypertension, there are few studies focusing on the role of adducin in tumor. Previous studies showed that adducin played a role in the evolution and progression of neoplasm. This review makes a brief summary on the structure, function and mechanism of adducin and how adducin functions in tumorigenesis and metastasis.

## INTRODUCTION

Adducin is a ubiquitously expressed membrane-skeletal protein, which was firstly extracted from human erythrocyte in 1986 [1]. In 1991, Joshi and Gilligan found two adducin subunits named α adducin (ADD1) and β adducin (ADD2) [2], respectively. In 1995, Dong et al., reported the existence of γ adducin (ADD3) [3]. Adducin can be phosphorylated by PKA, PKC, and Rho-kinase. Besides, it can also bind to Ca2+/Calmodulin to exert functions. There is no doubt that abnormal phospho-adducin will disturb its function in cell proliferation, motility and signaling. Previous studies reported that adducin showed an abnormal level of expression or unusual phosphorylation in some tumors, suggesting that adducin may play a role in the evolution and progression in tumors.

### Structure of adducin

Adducin is believed to self-associate into heterodimers and heterotetramers, which are composed of either α/β or α/γ. Heterodimers are the main modality. Adducin is localized at spectrin-actin junctions. α adducin and γ adducin are found in most tissues, whereas β adducin is abundant only in erythrocytes and brain.

The adducin family consists of three closely related genes: *ADD1*, *ADD2*, and *ADD3. ADD1* has been mapped by positional cloning to human chromosome 4p16.3 [4]. Human *ADD2* is localized at chromosome 2p13-p14 [5], whereas *ADD3* is on chromosome 10q24.2-24.3 [6]. The sequences of α adducin and β adducin share 66% similarity at amino acid level, while γ adducin displays 60-70% sequence similarity with either α or β adducin.

It is demonstrated that all the three adducin proteins contain an N-terminal globular head domain, a neck domain and a C-terminal protease-sensitive tail domain. At the end of tail domain, there is a 22-residue MARCKS-related domain that has high homology to myristoylated alanine-rich C kinase substrate (MARCKS) protein. The MARCKS-related domain has clusters of lysine residues and is highly conserved among those three adducin subunits [1–3, 7–11].

### Function of adducin

#### Adducin is important for formation and stabilization of membrane cytoskeleton

Adducin preferentially binds to spectrin-actin at the tail domain. It can not only recruit spectrin to spectrin-actin to promote forming complicated meshwork but also bundle actin filaments [1, 9, 12–16]. Previous studies indicated that there were two linkages between membrane skeleton and lipid bilayer: band 3-ankyrin-β-spectrin and glycophorin C-protein 4.1-β-spectrin. William demonstrated that adducin formed a bridge of band-3-adducin-spectrin to consolidate the stabilization of the membrane [17–19]. Additionally, adducin can inhibit capping the fast-growing ends of actin filaments as an actin capping protein. In this way, adducin could prevent addition or loss of actin subunits and make it easier to bundle actin filaments [15, 16].

#### Adducin is involved in cell signaling and ionic transportation

Adducin is the substrate of PKA, PKC, Rho-kinase and it can be phosphorylated by those kinases to get involved in cellular signal transduction. Moreover, adducin has been demonstrated to exert influence on ionic transport. In particular, adducin is related to Na^+^ transportation, such as Na^+^-H^+^ exchange, Na^+^-Li^+^ reverse transfer and Na^+^-K^+^-Cl^-^ co-transport [9, 20, 21].

#### Adducin is essential for mitosis

Phosphorylation of ADD1 at Ser12 and Ser335 by cyclin-dependent kinase 1 (CDK1) enables ADD1 to bind to myosin-X (Myo10) and associate with mitotic spindles. The depletion of ADD1 results in distorted, elongated, and multipolar spindles, accompanied by aberrant chromosomal alignment [22, 23]. This phenomenon indicated that adducin played a crucial role in mitosis. Some studies revealed that ADD2 regulated structural stability and segregation of the chromosome as ADD2 bound to the chromosome in the mitotic metaphase. Furthermore, it is also found that adducin participated in meiosis of murine oocyte, suggesting that adducin may be indispensable in embryogenesis [24].

#### Adducin regulates cell motility

Phospho-adducin by Rho-kinase regulates membrane ruffling and cell motility [11]. ADD1 is dephosphorylated and mostly bound to spectrin in the membrane skeleton of the resting platelet at sites where actin filaments attach to the ends of spectrin molecules. After platelet activation, adducin that phosphorylated by PKC at Ser726 releases from the membrane skeleton concomitant with its dissociation from spectrin and actin, leading to the exposure of barbed actin filament ends which contributes to the actin assembly reactions in lamellar and filopodia [25–27]. PKCδ and phospho-adducin by PKCδ promote cell dissemination and migration [28]. Taken together, adducin is believed to play a role in regulating cell movement.

#### Adducin plays a critical role in cell-cell adhesion

It has been long recognized that ADD1 and ADD3 are enriched at intercellular junctions in cultured epithelial cells and simple mucosal epithelia *in vivo* [29]. Recent studies revealed that adducin promoted recruitment of β-spectrin so as to strengthen epithelial adherens junctions and tight junctions and antagonize stimulus-induced junctional disassembly. This novel function of adducin is important for remodeling of epithelial junctions and restoration of epithelial barrier [30].

### Activation mechanism of adducin

**Figure 1:**
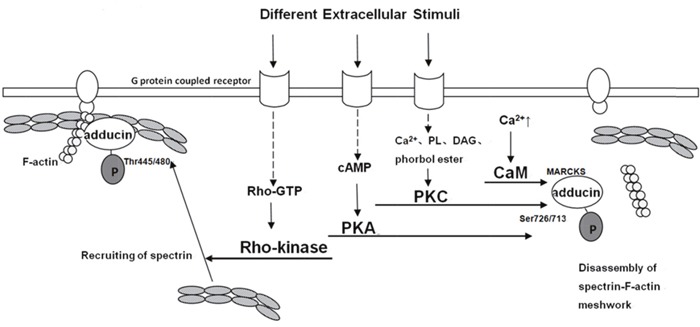
Model for different pathways of adducin phosphorylation [11, 26–36] A spectrin-F-actin meshwork is linked to the membrane by association with membrane accessory proteins such as band 4.1. Upon the activation of Rho-kinase by Rho during the action of extracellular stimuli, **α**-adducin is phosphorylated at Thr445 and Thr480. Phosphorylated **α**-adducin binds to F-actin and then facilitates the recruitment of spectrin to F-actin. This may promote the formation of a spectrin-F-actin meshwork beneath membrane. On the other hand, phosphorylated **α**-adducin at Ser726 by PKA or PKC, and at MARCKS through Ca^2+^/CaM inhibits the activity of adducin in recruiting spectrin to F-actin and functioning as an actin capping protein.

Post-translational modification of proteins is of vital importance in human body. Most common post-translational modifications include phosphorylation, ubiquitination, glycosylation, methylation and acetylation. However, few studies have reported that adducin could be modified by other ways except phosphorylation. Phosphorylation of adducin at specific sites by PKA, PKC and Rho-kinase is indispensable (Figure 1). One study mentioned that adducin was phosphorylated in the hepatocyte growth factor (HGF)/c-MET signaling pathway but not in detail [31]. It has also been demonstrated that adducin could bind to Ca^2+^/Calmodulin at the domain of MARCKs to exert functions. Following are four main activation processes.

#### Ca^2+^/Calmodulin pathway

The MARCKs-related domain of adducin has calcium-dependent binding of calmodulin. Different stimuli increase the concentration of intracellular calcium to trigger adducin bind to Ca^2+^/Calmodulin and hinder recruiting spectrin to spectrin-actin and capping the fast-growing ends of actin filaments [10, 30]. Calmodulin binding of native adducin and recombinant adducin polypeptides are inhibited by phosphorylation with PKC and by PKA. Other activities of adducin in association with spectrin-actin complexes are not affected by phosphorylation with PKC, which may indicate that the primary impact of phosphorylation by PKC is through modulation of the effects of calmodulin. Several observations showed that phosphorylation by PKC resulted in an almost complete loss of calmodulin binding for ADD2 and isolated peptide while only 50% reduction of binding to ADD1, suggesting that calmodulin preferentially binds to ADD2 when intracellular calcium is low while under higher levels of calcium it occupies binding sites of ADD1 as well as ADD2 [10].

#### cAMP-PKA pathway

Change in intracellular cAMP concentration is the main feature of this pathway. The production of cAMP, accelerated by activated adenylatecyclase (AC) contributes to the activation of PKA. Therefore, adducin, one downstream of PKA, is phosphorylated at Ser726 and Ser713 and it can also be targeted at Ser408, Ser436 and Ser481. Phospho-adducin inhibits the affinity of adducin and spectrin-actin and restrains from recruiting spectrin to actin [10, 11, 32].

#### PKC pathway

PKC isozymes is comprised of serine/threonine protein kinases that differ in cofactor requirement. For instance, the classical PKCs (α,β,γ) require a phospholipid (PL), diacylglycerol (DAG) or phorbol ester, and Ca^2+^ for optimal activation. The activation of PKCs (δ,ε,η,θ) is DAG, PL or phorbol ester dependent, but Ca^2+^ independent [29, 32, 33]. The phosphorylation of adducin at Ser 726 and Ser713 by PKC diminishes its interaction with actin and spectrin, hinders the recruitment of spectrin and exposes the barbed ends of actin filaments in motile cells [10, 26, 27, 32]. Recent studies elucidated that adducin was mainly targeted at Ser726 by PKCδ isozyme while PKCα may target at other sites except for Ser716 and Ser726 which remains unknown and requires further studies [28].

#### Rho/Rho-kinase pathway

Rho-kinase (ROCK) plays an important role in mitosis, muscle contraction, regulation of cytoskeleton, tumorigenesis and metastasis. Extracellular signals and some cytosolic proteins can affect the activity of ROCK. Extracellular stimuli (e.g. α-receptor agonist norepinephrine) aim at G protein-linked receptor and consequently activate Rho into Rho-GTP. The lateral binds the Rho binding domain of ROCK and leads to the exposure of ROCK's catalytic active center. Therefore, ADD1 is phosphorylated at Thr445 and Thr480 and phospho-adducin promotes its binding to F-actin and facilitates the recruitment of spectrin to F-actin to enhance the formation of a spectrin-F-actin meshwork beneath plasma membrane for membrane ruffling. It also regulates adducin to function as an actin capping protein [11, 35, 36]. However, previous studies haven't classified the role of Rho/Rho-kinase pathway in ADD2 or ADD3 phosphorylation.

### Advances in the study of adducin in tumorigenesis and metastasis

Phosphorylation is one of the most common post-translational modifications, whose target is mainly serine, threonine and tyrosine residues. The state of the downstream has changed between phosphorylation and dephosphorylation, bringing about changes in subcellular localization, life span and interaction with other proteins. As a result, phosphorylation is involved in cell growth, differentiation and apoptosis. Abnormal phosphorylation results in instability of proteins and unusual activity, thus contributing to abnormal cell proliferation, dysdifferentiation and apoptosis suppression in etiopathology. Abundant studies revealed that abnormal phosphorylation was related to tumorigenesis and the hereditary variation on kinase, phosphatase or substrate impacts human susceptibility to tumors.

Different activation pathways have different binding sites. As mentioned above, PKA and PKC target at Ser726 and Ser713 of adducin, while Rho-kinase works at Thr445 and Thr480. Abnormal activation of adducin is bound to have influence on cell proliferation, cell motility and signaling. For example, when S^716^ mutation occurs, ADD1 can be adhered to α nucleoprotein and then be transferred into the nucleus which lead to abnormality of cell communication and cell-cell adhesion [37].

So far, limited studies have shown changes in adducin levels and/or phosphorylation during cancer development and the effect on the motility and metastatic potentials of cancer cells. Different tumor types may share diverse changes in adducin level (Table 1). Specifically, in renal carcinoma, total adducin levels decreased while Ser-660-phosphorylated adducin levels increased, possibly due to enhanced protein kinase C δ activity in these tumors [38]. Adducin and PKCδ levels were increased in mammary tumor cell lines [39, 40]. ADD1 and ADD2 transcripts were found to be upregulated in basal cell carcinoma and squamous cell carcinoma when compared with the regular levels in normal keratinocytes [41]. In contrast, ADD3 was found to be downregulated in migrating glioma cells. Concerning that few studies have reported ADD2 in tumors, we mainly make a brief overview on ADD1 and ADD3 in tumorigenesis and metastasis from the following subparts.

**Table 1:** Differences of adducin in different tumors

tumor type	changes in adducin	authors and references
ovarian cancer	down-regulation of ADD1	Syed V [41]
non-cardia gastric cancer	ADD1 rs4963 C→G mutation	Wang MH [43]
colorectal cancer	ADD1 rs4963 C→G mutation	Shen N [44]
lung cancer	up-regulation of ADD1	Jen J [45]
small cell lung cancer	up-regulation of ADD1 and ADD3	Ma PC [31]
murine breast tumor	up-regulation of ADD3-Ia	Duretre M [46]
non small cell lung cancer	down-regulation of ADD3-Ib	Kwong LN [47], Langer W [48]
colorectal cancer	up-regulation of ADD3-Ia/ADD3-Ib	Tao M [49]
glioblastoma	down-regulation of ADD3	Mariani L [50], Rani SB [51]
T-lymphoblastic leukemia	*ADD3* fused with *NUP98*	Lahortiga L [52]

Note:ADD3-Ia or ADD3 (+14):encoded by variant 1 that expresses exon14; ADD3-Ib or ADD3 (-14):encoded by variant 2 and variant 3 that lose exon14

### ADD1 in tumors

It is worth noticing that adducin seems to function oppositely in different tumors. From the following mentioned studies, we can see that α adducin may serve as a tumor suppressor in some tumors (e.g. ovarian tumor) but a promoter in tumors like lung cancer. However, few studies have mentioned the exact mechanism of how α adducin works in tumor development.

#### ADD1 in ovarian cancer

By transcriptional profiling and stringent data mining criteria, one research identified that ADD1 expression were up-regulated by estradiol-17β (E2) in human ovarian surface epithelial but down-regulated by estrogen in ovarian cancer (OVCA) cells. It was also found that ADD1 overexpression in OVCA cells was associated with reduced cell proliferation, soft agar, colony formation and invasion, suggesting adducin functions as a tumor suppressor in ovarian cancer cells. Such a tumor suppressive function could be mediated via promotion of cell differentiation and suppression of mobility in the cancer cells. However, specific mechanism still remains unclear [42].

#### ADD1 in non-cardia gastric cancer and colorectal cancer

Recently, a missense variant at the codon of ADD1's phosphorylation site, rs4963 (Ser586Cys), is reported to modify the risk of non-cardia gastric cancer and colorectal cancer (CRC). The *ADD1-rs4963* C allele is at the codon of TCT encoding serine, whereas the G allele is at the codon of TGT encoding cysteine. When C→G mutation occurs, the serine residue changes into a cysteine residue, which cannot be phosphorylated due to lack of hydroxyl groups. Wang et al., conducted a case-control study with 1998 cases and 2008 controls and found that *ADD1-rs4963* might be associated with susceptibility to non-cardia gastric cancer [43]. Likewise, Shen et al., also performed a case-control study with a total of 1054 CRC cases and 1128 matched controls and also found that *ADD1-rs4963* might modify the risk of CRC [44]. It is speculated that the G risk allele of *ADD1-rs4963* might affect the phosphorylation of ADD1 and disturb its activity, including cell proliferation and communication, resulting in the development of non-cardia cancer and CRC [43, 44].

#### ADD1 in lung cancer

One study found that HGF and c-MET were overexpressed in small cell lung cancer (SCLC) and ADD1 was one of the downstream of HGF/c-MET signaling pathway [31]. Jen et al., observed that Zinc finger protein 322A (ZNF322A) was overexpressed in Asian and Caucasian lung cancer patients. Quantitative proteomic analysis revealed potential ZNF322A-regulated downstream targets, including ADD1, cyclin D1 (CCND1) and p53. They found that ZNF322A formed a complex with c-Jun and cooperatively activated *ADD1* and *CCND1* but repressed *p53* gene transcription. Reconstitution experiments indicated that CCND1 and p53 were important to ZNF322A-mediated promotion of cell proliferation, whereas ADD1 was necessary for ZNF322A-mediated cell migration and invasion, suggesting ADD1 was involved in tumorigenesis and metastasis of lung cancer [45].

### ADD3 in tumors

Apart from ADD1, some studies have reported the connection of ADD3 with tumors. ADD3 gene can be cut into three spliceosomes named variant 1, variant 2 and variant 3 and encode two kinds of proteins: one is ADD3-Ia encoded by variant 1 that expresses exon14 (ADD3(+14)); the other is ADD3-Ib encoded by variant 2 and variant 3 that lose exon14 (ADD3(-14)). Dutertrel observed that ADD3 (+14) was overexpressed in murine breast tumor of high degree of metastasis [46]. Similarly, ADD3 (-14) has been found to be down-regulated in non-small cell lung cancer compared with normal lung tissue [47, 48]. Tao's experiment showed that the expression levels of ADD3 and ADD3-Ib decreased in CRC tissues as compared with the normal mucosa while ADD3-Ia/ADD3-Ib ratio increased in CRC tissue. In contrast, reduced expression of ADD3 and its splicing isoforms have been observed in colorectal polyps, suggesting that changes in ADD3 and its isoforms expression in polyps are the early stage of CRC, leading to the report of the change of ADD3-Ia/ADD3-Ib ratio useless. As polyps developed into CRC, ADD3 (+14) increased to normal level and ADD3-Ia/ADD3-Ib ratio changed from normal level to a higher level, which conformed to the development of CRC. Besides, the expression changes of ADD3 and its splicing isoforms in the CRC may be relevant to its invasion ability [49].

Besides, Rani et al., found a lower level of ADD3 in metastatic glioblastoma cells. Their study demonstrated that miR-145 acted as a tumor-suppressor in glioblastoma since it apparently reduced proliferation, adhesion and invasion of glioblastoma cells, apparently by suppressing the activity of ADD3 and oncogenic protein Sox9 [50, 51]. In SCLC, ADD3 was increased due to overexpression of HGF and c-MET [31]. Recently, *ADD3* gene has been reported for the first time as a translocation partner of the nucleoprotein 98 gene (*NUP98*) in acute T-lymphoblastic leukemia [52]. However, none of those studies has mentioned how ADD3 exerts its function.

Taken together, we conclude that adducin has the following main functions relevant to tumor. Firstly, adducin promotes the ability of tumor cellular proliferation, migration and invasion. Secondly, adducin may function as a tumor suppressor in some tumors, such as in ovarian epithelial tumor and glioblastoma. Besides, adducin has different splicing isoforms that are differentially expressed in different stages of tumor progression. It is proved that differences exist in the phosphorylation sites of adducin in different tumors. For instance, phospho-adducin at Ser660 is at a high level in renal tumor. However, previous studies haven't revealed the mechanism of adducin in tumorigenesis. Considering the mechanism of adducin activation and its biological effects, we suggest that imbalance of phospho-adducin and dephospho-adducin, and overexpression or down-regulation of adducin lead to instability of membrane skeleton, abnormal formation of chromosomes, loss of cell-cell adhesion or reinforced adhesion and unusual membrane ruffling, which ultimately result in abnormalities in differentiation, proliferation, migration and invasion. However, detailed mechanism requires further studies.

### Prospects

Tumor is caused by abnormal cell proliferation from mutual interaction between heredity and environment. It is able to invade para-tissues and migrate to tissues far away from primary lesion through direct dissemination, lymph node metastasis, blood metastasis and implantation metastasis. However, the mechanism of tumorigenesis and metastasis in most tumors remains unknown. So far, adducin has not been reported to be modified by other ways except phosphorylation. Whether adducin has other modifications like ubiquitination and methylation remains unclear. It also interests us how other modifications may affect the phosphorylation of adducin and act on tumorigenesis. The splicing isoforms of adducin might be used as a biological marker for prophylaxis of tumor and a therapeutic target. Considering diverse phosphorylation sites of adducin in different tumors, whether there exists a phosphorylation site that expresses similarly in different tumors so it can be a targeted point to hinder the development of tumors. Additionally, given that adducin is not only involved in formation of the membrane cytoskeleton and cell motility and can also function as a tumor suppressor, the ability of adducin to control malignant biological behaviors worth studying. Nevertheless, all these hypotheses need further studies.
